# Phthalates and Alternative Plasticizers in Female Reproductive Health and Pregnancy: From Epidemiological Evidence to Nuclear Receptor-Mediated Mechanisms: A State-of-the-Art Review

**DOI:** 10.3390/biom16081109

**Published:** 2026-07-29

**Authors:** Audrey Antoine, Coline Charnay, Denis Gallot, Régine Quinard, Geoffroy Marceau, Corinne Belville, Lauren S. Richardson, Loïc Blanchon, Ramkumar Menon, Vincent Sapin

**Affiliations:** 1 iGReD, Team “Translational Approach to Epithelial Injury and Repair”, UMR6293 CNRS-U1103 INSERM, Université Clermont Auvergne, 63000 Clermont-Ferrand, France; audrey.antoine63@hotmail.com (A.A.); ccharnay@chu-clermontferrand.fr (C.C.); dgallot@chu-clermontferrand.fr (D.G.); rquinard@chu-clermontferrand.fr (R.Q.); gmarceau@chu-clermontferrand.fr (G.M.); corinne.belville@uca.fr (C.B.); loic.blanchon@uca.fr (L.B.); 2 Obstetrics and Gynecology Department, CHU Clermont-Ferrand, 63000 Clermont-Ferrand, France; 3 Biochemistry and Molecular Genetics Department, CHU Clermont-Ferrand, 63000 Clermont-Ferrand, France; 4 Division of Basic Science and Translational Research, Department of Obstetrics and Gynecology, University of Texas Medical Branch (UTMB), Galveston, TX 77555, USA; lestaffo@utmb.edu (L.S.R.); ra2menon@utmb.edu (R.M.)

**Keywords:** phthalates, alternative plasticizers, nuclear receptors, female reproductive health

## Abstract

Phthalate esters (PAEs), which are widely used as plasticizers in various products, are recognized as endocrine-disrupting chemicals (EDCs) that can affect female reproductive health. Numerous studies linked PAE exposure to several health hazards, including disruption of folliculogenesis, steroidogenesis, implantation, and pregnancy maintenance, contributing to complications such as miscarriage, preeclampsia, and preterm birth. In response to growing concerns regarding their toxicity, alternative plasticizers (APs) have been introduced, with particular attention given to the commonly used compounds, including di(isononyl) cyclohexane-1,2-dicarboxylate (DINCH), tris(2-ethylhexyl) trimellitate (TOTM), di(2-ethylhexyl) adipate (DEHA), and acetyl tributyl citrate (ATBC). While these alternatives are often presented as safer, recent studies have suggested that these compounds can also disrupt hormonal pathways and fertility regulation mechanisms. Both PAEs and APs have been shown to interact with key nuclear receptor systems, especially with peroxisome proliferator-activated receptors (PPARs) and steroid hormone receptors. These receptors are essential regulators of ovarian function, placental development, and pregnancy maintenance. Disruption of these signaling pathways, particularly in the placenta and fetal membranes, may contribute to altered inflammatory responses, impaired endocrine regulation, and increased risk of adverse pregnancy outcomes. This review provides a state-of-the-art synthesis of the current literature on PAEs and APs, focusing on exposure patterns, associations with female reproductive health and pregnancy outcomes, and the identification of dysregulated biological pathways. This review emphasizes the urgent need to limit plasticizer exposure, especially among women of reproductive age, in order to preserve reproductive health and prevent associated complications.

## 1. Introduction

Environmental exposure to endocrine-disrupting chemicals (EDCs) has become an important public health concern. This concern is particularly important during pregnancy, a vulnerable time for the mother and the developing fetus. Among these chemicals, phthalate esters (PAEs), alternative plasticizers (APs), and their metabolites are commonly detected in different human tissues, highlighting the urgent need to understand their potential effects on reproductive health. A literature search was conducted using the PubMed database to identify relevant English-language publications from 2000 to 2025. The search strategy included combinations of keywords such as “phthalates”, “alternative plasticizers”, “female fertility”, “pregnancy outcomes”, “nuclear receptors”, “placenta”, and “fetal membrane”. This review encompasses epidemiological, in silico, in vitro, and in vivo studies that analyze the effects of PAEs and APs on female fertility and pregnancy outcomes.

A primary mechanism underlying the effects of PAEs and APs is the disruption of nuclear receptor signaling pathways, which serve as key regulators of reproductive physiology. These receptors modulate trophoblast function, placental development, fetal membrane integrity, inflammatory responses, and hormonal metabolism. Consequently, dysregulation of these pathways by PAEs and APs represents a significant aspect of endocrine disruption with important implications for female reproductive health. This review will further examine this topic in Section 4, which addresses nuclear receptor-mediated mechanisms, focusing on the placenta and fetal membranes, to assess whether emerging plasticizers represent safer options. 

### 1.1. Phthalates

Phthalate esters (PAEs) are a large group of additives found in plasticized materials. PAEs were first introduced in 1920, and their production significantly increased during the 1950s with the rise of polyvinyl chloride [1]. Between 2007 and 2017, the global production of phthalates increased from 2.7 to approximately 6 million tons per year [2]. Phthalates are used in a wide range of objects and materials (e.g., food packaging, cosmetics, and toys) due to their physicochemical properties (colorless, flexible, and durable) [1]. In the medical field, phthalates are used in blood bags, catheters, and plastic tubing, and in the pharmaceutical industry, as excipients in certain extended-release medications [3,4]. PAEs are primarily classified into low-molecular-weight (LMW) and high-molecular-weight (HMW) compounds based on the length of their alkyl side chains. HMW PAEs, such as di(2-ethyl-hexyl) phthalate (DEHP), di-isodecyl phthalate (DiDP), di-isononyl phthalate (DiNP), di-octyl phthalate, butyl benzyl phthalate (BBzP), mono-isodecyl phthalate (MiDP), and mono(2-ethylhexyl) phthalate (MEHP), with side chains of ≥5 carbon atoms, are more stable and less hydrophilic than LMW PAEs [5] In contrast, LMW PAEs, such as di-butyl phthalate (DBP), di-ethyl phthalate (DEP), and dimethyl phthalate, have side chains of one to four carbon atoms and can be easily hydrolyzed into their monoester forms in nature (Table 1) [6,7]. This classification underpins differences in physicochemical properties, industrial applications, environmental behavior, and toxicological profiles between the two groups. HMW phthalates are predominantly used as plasticizers in polyvinyl chloride (PVC) products, such as medical devices, food packaging, and construction materials, whereas LMW phthalates are mainly found in personal care and pharmaceutical products. Among these, DEHP, DBP, BBzP, and DiNP are the most extensively studied in toxicological research due to their widespread human exposure and the substantial body of toxicological evidence regarding their endocrine and reproductive effects.

PAEs are weakly bound to plastic matrices through covalent interactions, allowing them to migrate easily into the environment and eventually enter the human body [8]. The primary route of phthalate contamination is through the ingestion of contaminated food [9], depending on their solubility in the polymer matrix [10,11]. Other exposure routes include inhalation, transdermal absorption, and intravenous exposure.

After absorption, phthalates are metabolized through hydrolysis, oxidation, and conjugation reactions, generating a variety of metabolites whose relative abundance depends on the parent compound’s physical characteristics. Low-molecular-weight phthalates are primarily hydrolyzed into monoesters, whereas high-molecular-weight compounds undergo extensive oxidation. In the case of DEHP, the primary metabolite MEHP is further oxidized into secondary metabolites as mono(2-ethylhexyl) phthalate (MEHHP), mono(2-ethyl-5-oxohexyl) phthalate, or mono(3-ethyl-5-carboxypentyl) phthalate (MECPP), which are the predominant urinary biomarkers in humans. These metabolites are excreted primarily in urine and, to a lesser extent, via feces, making urinary metabolites widely accepted biomarkers of phthalate exposure [12].

While PAEs are considered non-persistent pollutants, their constant presence indicates that populations are consistently exposed to them [13,14]. Investigations of their mechanisms of action have shown that phthalates are endocrine disruptors (EDs), leading to harmful effects on adults and their offspring [15,16]. In response to legal restrictions on their use in the plastic industry, various alternative plasticizers have been developed and are currently used.

### 1.2. Alternative Plasticizers (APs)

Unlike phthalates, APs do not follow a standardized classification system (Table 1). Instead, they are categorized according to their chemical structure into several groups, including citrate (acetyl tributyl citrate, ATBC), cyclohexane dicarboxylates (diisononyl cyclohexane-1,2-dicarboxylate, DINCH), adipates (di(2-ethylhexyl) adipate, DEHA), terephthalates (di(2-ethylhexyl) terephthalate, DEHTP), and trimellitates (tris(2-ethylhexyl) trimellitate, TOTM) [17].

While many of these APs share structural similarities with PAEs, they generally exhibit lower migration into food and present safety profiles that raise fewer concerns regarding endocrine disruption and their genotoxicity or carcinogenicity [18,19]. Consequently, APs have been increasingly adopted as phthalate substitutes in a wide range of applications, including medical devices, toys, and food containers. This substitution is largely driven by regulatory restrictions on several high-molecular-weight phthalates worldwide. Despite their widespread use, APs are not subject to a harmonized regulatory framework, and their regulatory status varies among countries and applications [20].

Among alternative plasticizers, DINCH has emerged as one of the most used substitutes for HMW phthalates and, consequently, has become a major focus of recent research. Although initially considered a safer alternative, experimental studies suggest that DINCH may exhibit biological activity, including effects on endocrine pathways.

## 2. Epidemiology

Plasticizer exposure has been widely evaluated through human biomonitoring studies. These investigations quantify PAEs, APs, and their metabolites in biological matrices such as urine and/or blood. For example, the analysis of monoester metabolites enables researchers to accurately assess phthalate concentrations in the general population while minimizing the risk of contamination by external influences.

### 2.1. General Population

#### 2.1.1. Phthalates and APs in Urine

Numerous studies quantified PAE metabolite concentrations in urine samples collected across Europe [21,22,23], Asia [23,24,25], and North America [23,26,27]. In total, 22 metabolites have been detected including mono-methyl phthalates (MMP), mono-ethyl phthalate (MEP), mono-n-butyl phthalate (MBP), mono-benzyl phthalate (MBzP), Mono-isobutyl phthalate (MIBP), mono-(2-ethylhexyl) phthalate (MEHP), Mono-(2-ethyl-5-hydroxy-hexyl) phthalate (MEHHP), Mono-(2-ethyl-5-oxo-hexyl) phthalate (MEOHP), Mono-(2-ethyl-5-carboxy-pentyl) phthalate (MECPP), and Mono-(2-carboxymethyl-hexyl) phthalate (mCMHP), with similar concentration ranges of 1–200 µg/L. A comparative analysis suggested a considerable worldwide decline in phthalate concentrations from 1990 to 2015, with, for example, a decrease by a factor of 2 of MBzP and DEHP metabolite concentrations from 2002 to 2008 [28,29,30].

Urinary metabolites of alternative plasticizers, including DINCH derivatives (OH-MINCH and oxo-MINCH), as well as metabolites of other substitutes, have been increasingly used as biomarkers of human exposure in epidemiological studies [31,32,33]. For example, cyclohexane-1,2-dicarboxylic acid monoisononyl ester (cMINCH) has been detected in urine samples from children, adolescents, and adults across Europe and North America [28,34,35]. Over the past 20 years, DINCH metabolite levels have steadily increased worldwide, reflecting its progressive replacement of regulated PAEs. In the United States, they were undetectable in 2000 but reached rates of 16% in 2007 and 21% in 2012, with similar trends observed in Europe [31,32]. Comparable temporal increase was noted for DEHTP, with detection rates rising by 8% in 2024. In 2017, quantifiable DEHTP metabolites were detected in urine samples of young German adults, with a median concentration of 3.35 µg/L [36]. Finally, a study conducted in Korea in 2015–2016 found DINCH, DEHTP and DEHA metabolites in 68%, 40%, and 53% of the adult women samples, respectively [37].

#### 2.1.2. Phthalates and APs in Blood

In contrast to urinary biomonitoring, blood-based measurements provide evidence of systemic internal exposure to PAEs and, to a much lesser extent, alternative plasticizers. A study from Asia found that all serum samples contained MMP, MEP, MBP, MEHP, and MECPP, with detection rates of 79% to 100% and concentrations ranging from 0.70 to 61 µg/L [38]. MMP, MEHP, and MBP were the predominant phthalates in serum, with the highest concentration for MMP (3.4 ng/mL), followed by MEHP (3.3 ng/mL) and MBP (2.8 ng/mL). In Europe, MMP, MEP, MBP, MBzP, MIDP, and MEHP were found in more than 80% of serum samples [39]. Overall, these findings indicate that phthalates are bioavailable in the human population.

However, to date, no studies have quantified AP concentrations in serum or plasma, highlighting a major gap in exposure characterization at the systemic level. Emerging evidence indicates that exposure to plasticizers may begin in utero. Pregnancy is a critical period for fetal development, during which maternal exposure to environmental pollutants can negatively impact fetal health [40]. In utero exposure occurs through maternal transfer, triggering investigations of PAEs and APs in various matrices from pregnant women.

### 2.2. Pregnant Women

#### 2.2.1. Phthalates and APs in Maternal Urine

MEP is the predominant phthalate metabolite detected in maternal urine; however, other metabolites, including MnBP, DEHP metabolites (MEHP, MEHHP, MEOHP, and MECPP), MBzP, and MiBP, have also been identified [41,42,43,44]. Urinary PAE concentrations tend to increase throughout pregnancy, with higher concentrations of MEP, MiBP, MnBP, DBP, and other low-molecular-weight PAEs observed in the third trimester [41].

Urinary biomarkers of APs have also been reported in pregnant women. In Asian cohorts (Korea and Thailand), OH-MINCH was detected in 67.4% and 44.9% of urine samples, respectively, whereas MEHTP and MINCH were detected in less than 40% of samples [45]. DINCH metabolite concentrations in pregnant women are generally comparable to those reported in the general population [46,47,48]. In a French study, urine samples collected during the second and third trimesters showed DINCH metabolites (OH-MINCH and oxo-MINCH) in 99.8% of the samples, with a median concentration of 1.4 µg/L [49].

#### 2.2.2. Phthalates and APs in Maternal Blood

In a comparative study of two cohorts of pregnant women, six phthalate metabolites (MEP, MiBP, MnBP, MEHP, MiNP, and MiDP) were detected in more than 70% of the serum samples [50]. Similarly, a Chinese cohort study reported the presence of DEP, DBP, DEHP, MEHP, and MnBP in maternal blood samples [51]. In contrast, data on APs in maternal blood remain extremely limited. To date, no studies have been conducted on serum AP concentrations during pregnancy.

#### 2.2.3. Phthalates and APs in Cord Blood, Placenta, Amniotic and Follicular Fluid

Amniotic fluid (AF), collected via amniocentesis, represents another relevant matrix for assessing fetal exposure to PAE. In Europe, PAE metabolite detection frequencies in AF range from 15% to 100% depending on the country [42,52,53,54]. The major PAEs identified in AF are DINP metabolites (e.g., MIBP), DEHP metabolites (MEHP and MEOHP), MBzP, MEP, MBP, and MnBP, with concentrations ranging from 7.4 to 102.9 µg/L. In a U.S study, MEP, MBP, and MEHP were simultaneously detected in 18.5% of samples [12]. MBP was the most prevalent (92.6%), with a concentration of 263.9 µg/L, followed by MEP (39%) and MEHP (24%) at 9 µg/L and 2.8 µg/L, respectively. In contrast, studies conducted in Asia have reported lower detection rates, with MnBP, DEHP, and its metabolites (MEHP, MEHHP, and MEHP) present in 37.1% to 65.9% of AF samples [43,55]. One Iranian study reported MEHHP and MEOHP levels of 174.79 µg/mL and 5.03 µg/mL, respectively [56]. To date, no data are available regarding AF exposure to phthalate substitutes, such as DINCH, DEHTP, DPHP, and DEHA.

PAE compounds are also found in umbilical cord blood. Two studies in China reported the presence of the phthalate metabolites DNCP, DIBP, DBP, DCHP, DEHP, and DNP in cord blood samples [57,58]. One Italian study detected DEHP and its metabolite MEHP at concentrations of 0.52–1.19 µg/L in 88.1% of cord blood samples [59]. A study in the USA detected MBP, MBzP, MEP, and DEHP metabolites (MEOHP, MEHHP, and MECPP) in 92–100% of cord blood samples, with concentrations ranging from 6 to 25 µmol/L [60].

In addition, several phthalates were detected in placental tissue, including MEP, MBzP, and MCPP, as well as the sum of DnBP, DiBP, DEHP, and DiNP metabolites. This study also showed the presence of AP metabolites, with a median DEHTP metabolite concentration of 58.3 µg/mL and a sum of DINCH metabolites of 1.5 µg/mL [61].

**Figure 1:**
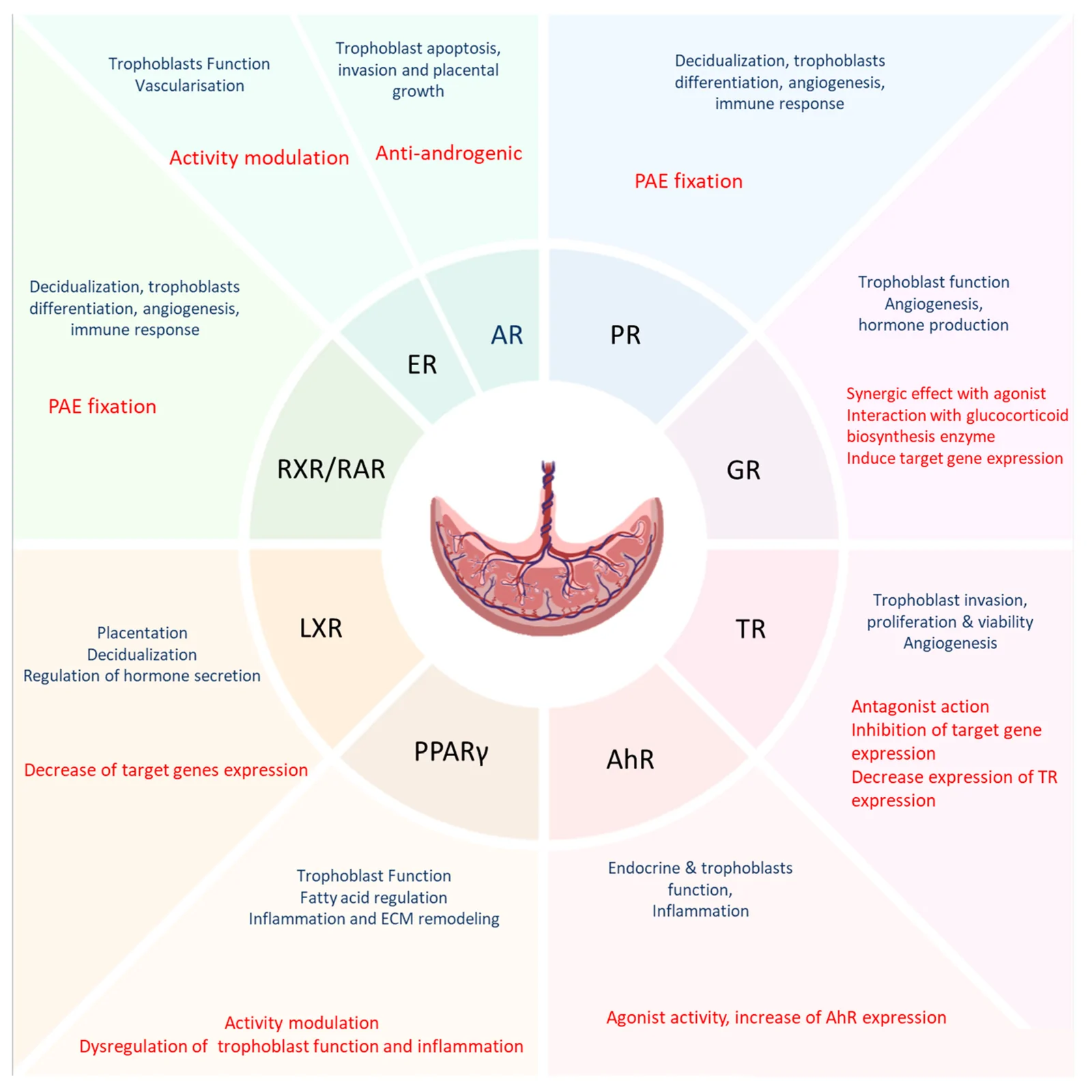
Involvement of nuclear receptors and perturbations by phthalates and alternative plasticizers. The roles of different nuclear receptors are highlighted in healthy states (blue) and exposure states (red).

Moreover, exposure may begin even before conception with PAE contact with the ovaries and oocytes via follicular fluid contamination. In a Chinese study, phthalate metabolite concentrations were measured in the follicular fluid of infertile women, and these metabolites were found in all samples [62]. In another study from China, the major metabolites detected were MEHP, MEHHP, MMP, MEP, MBzP, and MBP [63]. Together, all the reported studies suggest that pregnant women are exposed to PAEs and APs throughout pregnancy, and that these compounds can affect reproductive tissue, impair conception, contribute to pregnancy-related pathologies, and influence fetal development (Figure 1).

## 3. The Effects of PAEs and APs on the Female Reproductive Tract and During Pregnancy

The female reproductive tract has emerged as a major target of endocrine-disrupting chemicals (EDCs), with growing evidence linking exposure to these compounds to the development of several reproductive disorders. Moreover, the detection and accumulation of diverse EDCs, including bisphenol A and, more recently, microplastics, in serum and follicular fluid underscore the susceptibility of the ovarian microenvironment to environmental contaminants. Among these chemicals, PAEs and APs have attracted increasing attention due to their widespread exposure and their potential effects on female reproductive function [64,65,66].

### 3.1. Ovaries

The ovaries are essential for follicular development and steroid hormone production, and any disruption to these processes can have severe repercussions on reproduction [67,68]. In vivo, DEHP, DBP, BBzP, or DINP inhibit antral follicular development, which reduces the primordial follicle pool and total count, leading to decreased mature oocyte production and pregnancy rate reduction [67,69,70]. Additionally, DEHP disrupts oocyte maturation and activation before fertilization by inhibiting meiotic maturation and increasing oxidative stress [71,72]. Furthermore, PAEs decrease aromatase synthesis, a key enzyme in steroidogenesis, thereby suppressing estradiol production in the ovaries and may contribute to impaired ovulation [67]. In women, PAE exposure has been associated with the development of reproductive diseases such as premature ovarian insufficiency and polycystic ovary syndrome, which may lead to infertility [73,74].

Emerging evidence also suggests that APs, such as DINCH and DEHTP, may also interfere with steroid hormone pathways. Notably, one study showed that DEHTP metabolites had a more pronounced effect on steroidogenesis than DEHP metabolites, by increasing estrogen synthesis [75].

### 3.2. Uterus and Endometrium

The uterus plays a crucial role in zygote implantation and embryonic development, processes regulated by steroid hormones (estrogen and progesterone), which promote endometrial cell proliferation, differentiation, and angiogenesis [76]. Animal and epidemiological studies have shown a positive correlation between phthalate exposure and uterine dysfunction [77], including possible associations with fibroids and endometriosis [78,79]. However, current evidence does not establish a clear cause-and-effect relationship. In vitro, DEHP and MEHP increase cell viability and stress resistance, increase matrix metalloproteinase activity (MMP-2 and MMP-9), and stimulate ERK phosphorylation, all of which are associated with enhanced endometrial cell invasiveness, potentially contributing to the development of endometriosis [80,81]. Both fibroids and endometriosis are associated with embryo implantation failure, potentially due to reduced estrogen/progesterone levels, altered steroid receptor expression and decreased E-cadherin via MAPK and nuclear factor-_Κ_B pathways [82,83,84]. Phthalates also disrupt implantation by activating MAPK signaling, increasing susceptibility to oxidative stress, impairing trophoblast proliferation, and disrupting hormone regulation [85]. Beyond its impact on the female reproductive tract, PAE exposure persists throughout pregnancy, disrupting key molecular and hormonal pathways and may contribute to pregnancy complications affecting both maternal and fetal health.

### 3.3. Effects of Phthalates and Pregnancy

#### 3.3.1. Fertility

Studies investigating the impact of phthalates on female fertility, particularly in the context of in vitro fertilization, have reported an association between phthalate metabolites and impaired implantation, oocyte maturation, lower clinical pregnancy rates, and decreased live birth rates [86]. However, Høyer et al. (2018) reviewed the literature and found no association between DEHP exposure and delayed conception, highlighting the inconsistency of available evidence regarding the association between phthalate exposure and fertility [87]. In contrast, evidence regarding the effects of alternative plasticizers (APs) on female fertility remains limited. To date, the limited evidence currently available does not support an association with IVF outcomes. Specifically, MHINCH, a DINCH metabolite, has not been associated with intermediate or clinical in vitro fertilization outcomes [47].

#### 3.3.2. Miscarriage

Rodent (e.g., rat and mice) toxicology studies have shown that exposure to certain phthalates reduces embryonic survival, increases the resorption rate of implantation sites, decreases litter size, and raises the rate of spontaneous abortion [13]. Epidemiological studies have established an association between phthalate (MMP, MEP, MnBP and DEHP metabolites as MEHP, MEOHP and MEHHP) exposure and miscarriage [88] owing to decreased steroid hormone concentrations [89]. Discrepancies exist in the literature, with studies showing different results, especially a literature review by Radke et al., who concluded that evidence of an association between prenatal exposure to some phthalates and spontaneous abortion was limited [90].

#### 3.3.3. Preeclampsia and Intrauterine Growth Restriction

Maternal exposure to several phthalate metabolites (MBP, MMP, MEOHP, MEHHP, MEP, and MEHP) has been associated with morphological and functional placental impairments, including placental atrophy, reduced placental efficiency and weight, alteration of hormone production, and a reduced chorionic plate surface area [82,91]. At the cellular level, MEHP has been shown to induce oxidative stress, promote trophoblast apoptosis, and inhibit extravillous trophoblast invasion through dysregulation of matrix metalloproteinases (MMP-2 and MMP-9), enzymes essential for extracellular matrix remodeling during implantation and placentation [92,93]. From an epigenetic perspective, phthalates have been associated with alterations in DNA methylation and placental microRNA expression, potentially affecting trophoblast proliferation (by reducing epidermal growth factor receptor expression), apoptosis (by inhibiting the antiapoptotic gene Bcl-2) [74,92], and lipid metabolism (by inhibiting the cAMP/PKA signaling pathway) [94]. Phthalates also target membrane receptors such as P2X7, triggering downstream pro-inflammatory cascades via upregulation of pro-inflammatory cytokines and NLRP3 inflammasome activation, contributing to the systemic inflammation observed in preeclampsia [95,96]. Collectively, these molecular, cellular and epigenetic perturbations contribute to placental alterations leading to pregnancy complications such as preeclampsia, preterm birth and intrauterine growth restriction [97,98].

#### 3.3.4. Preterm Birth and Premature Rupture of Fetal Membranes

Epidemiological research demonstrated an association between prenatal exposure to phthalates, particularly DEHP metabolites (MEHP and MECPP) as well as MBP and MiBP and adverse pregnancy outcomes such as early pregnancy loss and preterm birth [99,100]. Conversely, other investigations identified a correlation between urinary concentrations of DEHP and MEHP and increased gestational duration [101,102]. Additional epidemiological evidence indicated links between exposure to PAEs detected in cord blood and maternal urine with elevated rates of preterm birth [90,103]. Several mechanistic pathways have been proposed to elucidate the potential relationship between phthalates and preterm labor. One mechanism involves the induction of intrauterine inflammation via cytokine production [59,104], which subsequently stimulates prostaglandin synthesis, leading to uterine contractions [105]. Another pathway implicates the disruption of uterine quiescence through activation of the hypothalamic–pituitary–adrenal axis in both fetus and mother, along with decidual hemorrhage and pathological uterine distension [106]. A third hypothesis attributes preterm birth to heightened oxidative stress resulting from PAE exposure, evidenced by elevated levels of oxidative stress markers such as 8-isoprostane [103,107]. Increasing evidence supports the notion that oxidative stress and corresponding DNA damage serve as critical mechanisms linking endocrine-disrupting chemical exposure to reproductive toxicity. Supporting this, recent studies examining exposure to per- and polyfluoroalkyl substances (PFAS) have reported significant associations between oxidative imbalance, lipid peroxidation, and compromised DNA integrity. Although conducted within the context of PFAS exposure, these findings reinforce the concept that redox dysregulation constitutes a common and fundamental pathway underlying EDC-induced reproductive toxicity and the pathophysiology of preterm birth [108].

In recent years, emerging evidence has suggested a potential association between alternative plasticizers and preterm birth. A Chinese study suggested that detectable urinary concentrations of MCOCH (a DINCH metabolite) were positively associated with preterm birth. However, these results should be interpreted with caution due to the low frequency of DINCH biomarker detection [14].

## 4. Nuclear Receptors

### 4.1. Introduction

Despite all these epidemiological correlations, the molecular mechanisms underlying PAEs and APs-induced adverse pregnancy outcomes remain poorly understood. Emerging evidence points to the disruption of nuclear receptor signaling pathways in gestational tissues, including the placenta and fetal membranes, as possible key factors. Nuclear receptors are intracellular transcription factors regulating numerous physiological processes, such as development, differentiation, reproduction, and metabolic homeostasis. There are 48 nuclear receptors classified into seven subfamilies, further divided into subgroups (class) according to their action and ligands.

### 4.2. Structure of Nuclear Receptors

Nuclear receptors are composed of six domains, named A to F [109,110]. The A/B domain, located in the N-terminal domain, corresponds to the ligand-independent transactivation domain (AF-1) involved in protein–protein interactions and cofactor recruitment for gene transcription. This domain varies in length and sequence among receptor subtypes, conferring their functional specificity. The C domain or DNA-binding domain comprises two zinc finger regions that enable sequence-specific DNA binding. The hinge region or D region is a conserved region linking the C region to the ligand-binding domain (called the E/F region). This domain contains nuclear localization signals and contributes to the recruitment of corepressors. The E/F region (located in the C-terminal part) mediates ligand binding, receptor dimerization, and transactivation through its ligand-dependent activation function 2 (AF-2). This domain is composed of 12 alpha helices (denoted H1 to H12) and a β-sheet. Ligand binding induces conformational changes in the receptor, allowing the AF-2 domain to interact with coactivators, thereby modulating the transcriptional activity of target genes.

### 4.3. Mechanisms of Action of Nuclear Receptors

All nuclear receptors share a conserved structural organization and common role in the regulation of gene transcription, although they differ in biomechanical mechanisms. In the steroid receptor family, ligand (hormone) binding induces dissociation of heat shock proteins, enabling receptor activation and subsequent translocation into the nucleus. The complex ligand-receptor binds to the response element composed of a palindromic sequence at upstream promoter sites. DNA binding is coupled with the recruitment of coactivator proteins (like steroid receptor coactivator SRC) and subsequent transcriptional activation. The class II family functions as a heterodimer present in the nucleus with the retinoic X receptor (RXR) and binds as a dimeric complex to direct repeat response elements. In the absence of a ligand, transcriptional activity is repressed through the recruitment of a corepressor complex to the DNA-bound heterodimer. In the presence of a receptor’s ligand, corepressor proteins are released, and coactivators are recruited, leading to transcriptional control [111,112]. Regarding orphan receptor function, current evidence indicates that these receptors can heterodimerize with RXR or bind as monomers at response elements to carry out their function.

## 5. Impacts of PAEs and AP on Nuclear Receptors in the Female Reproductive Tract: Focus on the Placenta and Fetal Membranes

Nuclear receptors constitute a signaling network that regulates endocrine, metabolic, inflammatory, and developmental processes essential for pregnancy maintenance [113,114,115]. In the placenta and fetal membranes, these receptors operate as an interconnected network rather than isolated pathways. Expression of nuclear receptors is both cell- and tissue-specific, and its expression dynamically changes across gestation.

Several docking and transactivation approaches suggest that PAEs and APs may interfere with this nuclear receptor network by acting as modulators of receptor activity [113,114,115] (Table 2).

### 5.1. Steroid Nuclear Receptors

Steroid nuclear receptors are central regulators of implantation, placental development, trophoblast differentiation, and fetal membrane integrity. PAEs and APs can interfere with these signaling pathways, leading to dysregulation of key reproductive processes.

#### 5.1.1. Estrogen Receptors

Estrogen receptors (ERs), ERα and ERβ, are encoded by distinct genes and display distinct but overlapping tissue distribution. In the placenta, ERα is expressed especially in villous trophoblasts (cytotrophoblasts and syncytiotrophoblasts) [116]. ERα isoform is essential for the development of secondary sexual characteristics in women, embryo implantation and ovarian function [117]. Furthermore, the β isoform is expressed in villous syncytiotrophoblasts and the amnion epithelium [116]. In the placenta, ER activation by its natural ligand estradiol (E2) plays a key role in trophoblast proliferation, cytotrophoblast differentiation into syncytiotrophoblasts and placental vascular development, all processes essential for expanding the maternal-fetal exchange surface [118,119,120]. The amnion contributes to estrogen production during pregnancy, and in vitro studies on the WISH amniotic cell line have shown that estrogen regulates inflammation responses and prostaglandin secretion [121,122].

In silico analyses have demonstrated that several phthalates and their metabolites bind to ER isoforms with varying affinity. Regarding ERα, DINP, DPHP, BBzP, MPhP, and DnOP have higher docking scores than the natural ligand estradiol. In contrast, ERβ binds MphP, MEPH, and MnHP, though with lower affinity than the natural ligand [113]. Additional docking studies have identified other phthalates, such as BBP, DCHP, DBP, and DIBP, as weak ligands for ERs, with some exhibiting selective estrogen receptor modulator (SERM)-like properties [114]. Several phthalates (DcHP, BBEP, and DEHP) also show anti-estrogenic activity on ERβ [115,123]. In vitro assays have shown that phthalates such as DEP, DIBP, and DBP can induce ERα activity, with DBP showing the highest affinity for ERα [124,125]. Collectively, these findings indicate that phthalates can act as weak agonists or antagonists, potentially interfering with estrogen-dependent signaling pathways. Alternative plasticizers such as DINCH metabolites (M2NCH, OH-MINCH, and oxo-MINCH) can activate ERα. However, DINCH itself and all its metabolites (M2NCH, MINCH, OH-MINCH, oxo-MINCH, and cx-MINCH) generally exhibit antagonistic activity toward ERα. Most also act as antagonists of ERβ, except for M2NCH, which shows agonist activity at 100 µM [126].

#### 5.1.2. Androgen Receptors

Androgen receptors (AR), α and β, are widely distributed in reproductive tissues (breast, ovaries, and uterus) and the nervous system [127,128]. In the placenta, AR regulates decidualization processes, trophoblast differentiation, angiogenesis, and immune modulation [129,130]. AR is expressed in cytotrophoblasts, syncytiotrophoblasts, stromal cells [131], and in the amnion during pregnancy [131]. Although the implications of AR in fetal membranes remain largely unexplored, the presence of the enzyme required for local androgen synthesis suggests a potentially significant, yet understudied, function of AR signaling in these tissues [132].

Structural interactions between AR and phthalates have shown that DEHP and its metabolites (MEHP, 5-OH-MEHP, 5-oxo-MEHP, 5-cx-MEPP, and 2-cx-MMHP) present a strong affinity for this receptor [133]. In vitro studies have demonstrated that DBP, MBP, and DEHP display agonist properties, which can be inhibited by co-treatment with an AR antagonist [134]. However, these findings are not consistently reproduced, as other studies have reported no agonist activity for these compounds on AR signaling [115,125,135]. Engel et al. found that phthalates showed no androgenic activity, while identifying anti-androgenic effects of DiBP, DBP, DcHP, BBeP, DPeP, DiHP, and DHP [115]. These discrepancies may be attributed to differences in experimental design, including distinct reporter gene systems and cell lines. Regarding APs, DINCH and its metabolites do not directly activate AR. Nevertheless, most DINCH metabolites, with the exception of DINCH itself and MINCH, significantly enhance AR activation in the presence of an exogenous agonist. These findings suggest that certain DINCH metabolites may exert modulatory or synergistic effects on AR signaling pathways rather than acting as direct AR agonists [126].

#### 5.1.3. Progesterone Receptors

In humans, PR-A, PR-B, and PR-C isoforms are expressed in several reproductive tissues, from a single gene using distinct estrogen-inducible promoters [136]. In women, PRs are involved in sexual behavior, neuroendocrine gonadotrophin regulation, fertility, mammary gland cell differentiation and influence cardiovascular and cognitive processes during pregnancy [137,138]. Specifically, PR-A loss results in implantation failure and impaired decidualization [139]. In the trophoblast models (HTR8/SVneo), progesterone promotes trophoblast growth and invasion by modulating apoptosis-related pathways via the increase of antiapoptotic BCl-2 protein expression and decreasing pro-apoptotic FAS-L, Fas, caspase-3, and caspase-8 protein expression [140]. In fetal membranes, PR isoforms are expressed at relatively low levels in both amnion and chorion [141].

Docking studies have shown that MIDP, DnOP, BBZP, DPhp, and DEHP and its metabolites can bind to their ligand-binding domain but with lower affinity than natural ligands [142,143].

#### 5.1.4. Glucocorticoid and Mineralocorticoid Receptors

The glucocorticoid receptor (GR) plays a major role in reproductive function and is expressed throughout pregnancy in the placenta, uterine stroma, and fetal membranes [144]. In the placenta, glucocorticoids regulate trophoblastic invasion, differentiation, and proliferation [145]. They also reduce angiogenesis and placental hormone production, increasing stromal vascular permeability during implantation and present anti-inflammatory properties [146,147,148,149]. In fetal membranes, GR is expressed in the amnion and trophoblastic cells in the chorion [150,151]. Within the amnion, glucocorticoids contribute to extracellular matrix remodeling and regulation of prostaglandin synthesis through GR-mediated pathways [152,153]. Regarding mineralocorticoid receptors (MR), they are expressed in the syncytiotrophoblasts, cytotrophoblasts, and interstitial cells of the villous core [154] where they are involved in placental growth, trophoblastic proliferation, and fetal development [155]. Moreover, MR signaling regulates placental sodium transport, which is crucial for maintaining uteroplacental blood flow and, consequently, normal fetal development [155,156].

A docking study demonstrated that several phthalates (MIDP, DEHP, MBzP, MINP, MPhP, DnOP, DCHP, and MCHP) displayed higher binding affinity than the endogenous ligand [157]. In addition, PAEs may interact with enzymes involved in the glucocorticoid biosynthesis pathway, suggesting a potential for pathway dysregulation [157]. In vitro evidence has shown that DEHP, DINP, DBP, DIBP, DEP, and dimethyl phthalate neither activate nor inhibit GR activity. Nevertheless, DEHP and DINP exhibit synergistic effects in the presence of dexamethasone, a GR agonist [158,159,160]. Likewise, MBP has been shown to promote GR nuclear translocation and enhance downstream target gene expression at both transcript and protein levels when co-exposed with a ligand [161]. Whether PAEs dysregulate the GR pathway remains unclear. However, in vivo studies in male mice suggest that DBP can increase testosterone production via GR-dependent mechanisms [162]. Although GR and MR share an endogenous ligand, most studies have focused on GR. In rat testis, in utero exposure to DEHP reduced adrenal aldosterone production, MR expression and MR target gene expression, leading to decreased testosterone levels [163,164]. Collectively, these findings suggest that PAEs may disrupt both GR and MR signaling pathways, potentially altering genes involved in stress responses and steroid hormone regulation. Further research is needed to clarify the precise mechanisms underlying PAE-mediated endocrine disruption.

### 5.2. Class II Receptors

#### 5.2.1. Retinoic Acid Receptors and Retinoid X Receptors

The “retinoic acid receptor” class comprises two families of nuclear receptors—retinoic acid receptor (RAR) and retinoid X receptor (RXR)—each consisting of three isoforms (α, β, and γ) and encoded by distinct genes. RXR isotypes (RXRα, RXRβ, and RXRγ) are encoded by distinct genes and present tissue-specific expressions and functions. All isotypes are activated by retinoids (activated derivatives of retinol) with a specificity of 9-cis retinoic acid for RXR. Furthermore, free fatty acids, such as docosahexaenoic acid, arachidonic acid, and eicosapentaenoic acid, are RXR ligands. RARs and RXRs play essential roles in development, decidualization, and placentation, as evidenced by knockout models in which deletion leads to embryonic lethality and placental defects [165,166]. In humans, the retinoid pathway is present in the placenta and fetal membranes, where RXRs regulate hormones that are essential for maintaining pregnancy, such as chorionic gonadotropin and leptin [167,168]. RXR expression in fetal membranes suggests potential heterodimerization with other receptors, such as liver X receptor (LXR) and the peroxisome proliferator-activated receptor (PPAR), which affects gene regulation in these tissues [169,170]. RARα and RARγ isoforms are expressed in the amnion and chorion within the fetal membranes, as well as in villous and extravillous trophoblast cells in the placenta [171,172].

A molecular docking study demonstrated that RXRs can bind various phthalates with isoform-specific binding preferences [173]. RXRα was able to bind to DEHP, DPHP, MNDP, BBZP, and MIDP, while RXRβ could bind to DEHP, MIDP, DINP, DNNP, DIDP, DPHP, DHP, MNDP, and BBZP. Furthermore, the gamma isoform was found to bind MBZP, DPHP, MIDP, MPHP, MINP, BBZP, and MEHP. The primary impact of phthalates on RXRs appears to arise from their ability to bind these receptors and modulate the transcriptional activity of RXR heterodimer partners, including LXR and PPARs. In contrast, little is known about PAE-mediated disruption of retinoic acid receptor (RAR) signaling, and no molecular docking studies have yet evaluated PAEs in relation to RAR. However, phthalates with a C4 alkyl ester chain have been shown to inhibit retinoic signal up to 40% in embryonic cells [174]. Further research is required to understand the potential effects of alternative plasticizers on these receptors.

#### 5.2.2. Liver X Receptors (LXRs)

The LXR group comprises the two isotypes LXRα and LXβ, and they differ in distribution [175,176]. These receptors play major roles in fatty acid and phospholipid metabolism, cholesterol homeostasis, and insulin sensitivity [177,178,179], and they show anti-inflammatory properties [180]. LXRα and β are essential for ovarian function via steroid hormone regulation synthesis, cholesterol transport, and uterine quiescence [181]. Both isotypes are expressed in the placenta, where they regulate placentation, cholesterol transport, and biosynthesis of lipids and inhibit trophoblast invasion, syncytialization, and trophoblast differentiation at term [182,183,184,185]. Indirect LXR activity has also been observed in fetal membranes, with its target gene Abca1, expressed in amnion and chorion, exerting anti-inflammatory effects [186]. However, the specific role of LXR in fetal membranes remains to be investigated.

Molecular docking studies have shown that phthalates, such as DEHP, DIBP, DIDP, and DINP, can bind to LXRs. An in vitro study demonstrated that PAE exposure decreased the expression of LXRα and its target genes (SRBP-1 and LGK) [187]. In the liver, PAEs disrupt LXR, leading to lipid accumulation and increased pro-inflammatory cytokine expression (IL-6, IL-8, IL-1β, and TNF-α) [188]. Although no studies have specifically investigated LXR-phthalate interactions in the placenta or fetal membranes, MEHP has been shown to dysregulate LXR signaling in the ovaries, leading to a 40% increase in LXRα expression [189]. This finding suggests that phthalates could alter lipid/cholesterol pathways in the placenta and fetal. Further research on LXR dysregulation in the placenta due to phthalate exposure is required.

#### 5.2.3. Thyroid Receptors (TRs)

Human TR proteins are transcribed from two distinct genes, TRα and TRβ, which are involved in growth and development and in central nervous system development [190,191]. TRα1, α2, and β are expressed in all trophoblasts and the decidua [192,193,194]. Thyroid hormones affect villous and extravillous trophoblast proliferation, invasion, and viability in vitro by upregulating MMP-2, MMP-9, and MMP-3 expression. They also reduce apoptosis in extravillous trophoblasts, supporting their invasive phenotype [192,195,196,197,198]. Moreover, thyroid hormones are involved in the angiogenesis of placental and decidual tissue by increasing the expression of vascular epidermal growth factor and inflammatory cytokines [199,200,201]. No studies have detected TR in fetal membranes, and its involvement in the physiology of these membranes remains to be investigated.

In silico assays have demonstrated that DEHP and its metabolites can bind to TRα1 and TRβ1 [202]. The metabolite 5-OH-MEHP shows agonist activity and can act synergistically with thyroid hormones. In vitro studies have shown that phthalates, such as DEP, DEHP, BBP, DBP, and DCHP, act as TRβ antagonists and inhibit its target gene expression [134,203]. In the placenta, DEHP further impairs the expression and nuclear translocation of TRβ target genes, such as vascular epidermal growth factor (VEGF), placental growth factor (PIGF), and insulin-like growth factor (IGF) [204]. In vitro studies have shown that DEHP decreases THRα and THRβ transcription in trophoblastic cells and reduces protein expression in JEG3 cells [205]. This disruption of thyroid hormone signaling may contribute to placental abnormalities and pregnancy complications, including intrauterine growth restriction. However, the underlying mechanisms remain unclear. Structural analyses have indicated that non-phthalate plasticizers (e.g., DINCH, acetyl tributyl citrate, and DEHA) can stably bind to the TRα ligand-binding domain [206]. Additionally, DEHTP and its metabolite MEHTP also display antagonist effects on TR.

#### 5.2.4. Xenobiotic Nuclear Receptors

Xenobiotic receptors, including constitutive androstane receptor (CAR), pregnane X receptor (PXR), and aryl hydrocarbon receptor (AhR), integrate environmental chemical exposure with endocrine, metabolism and immune regulation [207,208,209,210,211,212,213,214,215]. CAR and PXR have low expression in the placenta, and their function in this tissue remains unknown [216,217]. AhR is highly expressed in the human placenta particularly in syncytiotrophoblasts located at the periphery of the chorionic villi, where it plays a critical role in trophoblast invasion and migration, where it controls placental trophoblast invasion and migration [210]. Furthermore, AhR is also involved in fetal implantation by reducing the survival rate of implanted embryos [218,219]. In the placenta, AhR activation promotes inflammation via monocytes and macrophages stimulation, leading to the expression of pro-inflammatory cytokines (e.g., TNF-α, IL-1β, and IL-8) and their receptors [220,221,222,223,224,225,226]. This inflammatory response induces cyclooxygenase-2 expression in trophoblasts [227], enhancing prostaglandin synthesis and promoting uterine contractions [228]. Although AhR expression has not been confirmed in fetal membranes, the amnion responds to AhR ligand [229]. A possible mechanism of AhR-mediated weakening of the fetal membranes involves elevated inflammation and COX-2 expression, both key factors in membrane rupture.

In silico assays have demonstrated that 23 diester phthalates and 10 monoesters can bind to CAR [230]. Binding activity assays have further confirmed that most diester phthalates, except for DHP, DnOP, DnNP, DIBP, DINP, DIOP, DEHP, and DIDP, induce CAR activity. Among monoesters, MEP, MIBP, and TBMEHP show strong binding affinity [231]. Regarding PXR, in vitro studies have shown that DEHP, MEHP, and MBzP are PXR agonists, and DBP has the strongest effect on transcriptional control [59,232]. The xenobiotic metabolism pathways activated by CAR and PXR also process endogenous substrates, including those involved in steroid hormone metabolism, thereby contributing to endocrine disruption [212,233]. Regarding AhR, phthalates such as DEHP, DnBP, and DIBP increase AhR expression in placental samples [234]. In reproductive tissues such as the ovaries, MEHP increases AhR gene expression, while BBP induces necrosis in granulosa cells via an AhR-dependent mechanism [235]. AhR plays roles in xenobiotic metabolism, estrogen regulation, and placental vascularization [236,237,238], thus its dysregulation by phthalates could contribute to complications in pregnancy. Further research is required to clarify the effects of phthalates on AhR in placental and fetal membranes.

#### 5.2.5. Peroxisome Proliferator-Activated Receptors (PPARs)

The PPAR family comprises three isoforms, PPARα, PPARβ, and PPARγ, encoded by three different genes [176,239]. PPARα is involved in fatty acid oxidation, lipid metabolism, glucose homeostasis, and the development of insulin resistance. PPARβ is involved in wound healing, glucose homeostasis, and adipogenesis. PPARγ is involved in adipogenesis, lipid storage, glucose homeostasis, and inflammation [239]. The three PPAR isoforms are expressed in the placenta. PPARα and PPARβ are found in term placental syncytiotrophoblasts and villous trophoblastic cells. While PPARα knockout mice do not show placental abnormalities [240,241], PPARβ deficiency results in a reduced labyrinth zone and impaired placental-decidual attachment [242]. At term, PPARα expression remains stable in the amnion but decreases in the chorion, whereas PPARβ expression increases in both chorionic and amniotic regions [243]. PPARγ is the most extensively studied isoform in placental biology due to its critical physiological role, and its functions are discussed separately in detail below.

In silico, PPARα can bind to DINP, DEHP, DNOP, BBzP, DPHP, DIDP, and DHP, while PPARβ can bind to DIDP, DNDP, MnDP, BBZP, DEHP, DINP, MIDP, MBzP, and MPHP [173,244]. Transactivation assays have demonstrated that PPARα is activated by MEHP and MBzP in a concentration-dependent manner [245] and by MEHP, MINP, MnOP, and MIDP without inducing the expression of its target genes [246]. However, studies in other cell types, including adipocytes, hepatocytes, testis epithelial cells, and granulosa cells, have shown that PAEs can induce PPARα target gene expression (e.g., CD36, acyl-CoA oxidase, multifunctional protein II, Cyp4a, FABP4 and NDRG1) [247,248,249]. Moreover, PPARα expression is upregulated by MEHP in the placenta, suggesting that phthalates can dysregulate the PPARα signaling pathway [250]. While DINCH does not stimulate PPARα, its metabolites induce isoform-specific activity in a concentration-dependent manner. Recently, MINCH was reported to be a PPARα agonist at concentrations of ≥50 μM [126,251]. Studies in adipose tissue, liver, and testis cells have shown that DINCH alters PPARα expression and associated pathways [251,252]. These results suggest that the dysregulation of PPARα in diverse tissues could be due to PAEs, and the PPARα pathway could thus be altered in the placenta and fetal membranes. However, further studies are required to verify this possibility. Similarly, a range in the transactivation of mouse PPARβ by phthalate monoesters was also observed, but this effect was not found in humans [246].

#### 5.2.6. Peroxisome Proliferator-Activated Receptors γ (PPARγ)

The key role of PPARγ in fetal development has been demonstrated in PPARγ null mice, which exhibit embryonic lethality due to vascular defects in the placenta and impaired lipid metabolism, evidenced by the absence of lipid droplets [253]. In the human placenta, PPARγ expression varies across gestation [254,255]. During the first trimester, PPARγ is mainly localized in villous cytotrophoblasts and extravillous trophoblasts, and in the second trimester, it is detected in the columns of anchoring villi and cytotrophoblasts. During the third trimester, PPARγ is localized in villous syncytiotrophoblasts and in villous and extravillous cytotrophoblasts [182,256]. In fetal membranes, PPARγ expression is high and stable throughout gestation until the period before labor, when its expression decreases [243]. PPARγ has been identified as a regulator of trophoblast differentiation, migration, and invasion with ligand-dependent effects. The synthetic agonist troglitazone enhances cytotrophoblastic differentiation and migration [257,258,259], while its natural agonist (15dPGJ2) promotes trophoblastic apoptosis rather than differentiation [260]. PPARγ-mediated trophoblast differentiation is associated with the stimulation of hormone secretion, such as human chorionic gonadotropin, human placenta lactogen, human placental growth hormone, and leptin [261]. Activation of this receptor impedes syncytiotrophoblast invasion, whereas its inhibition facilitates this process [256], possibly through decreased availability of insulin-like growth factors, key promoters of trophoblast invasion [259,262]. This may involve reduced expression of pregnancy-associated plasma protein A (PAPP-A). Additionally, PPARγ upregulation via heme oxygenase-1 may further contribute to these inhibitory effects on trophoblast invasion [263]. One study showed that IL-17 activated PPARγ, promoting proliferation, migration, and invasion of HTR8/SVneo cells in vitro [264]. In primary trophoblast, PPARγ-induced differentiation is associated with lipid droplet accumulation in syncytiotrophoblasts, enhances fatty acid uptake and storage, through upregulation of fatty acid transporter protein (e.g., Fatty acid transport protein 1 and 4) expression [265,266]. PPARγ is also involved in controlling the inflammation process. PPARγ activation inhibits the secretion of pro-inflammatory cytokines (e.g., IL6, IL8, and TNF-α) and the degradation of extracellular matrix enzymes in the placenta, amnion, and chorion by repressing the nuclear factor-kB signaling pathway [262,267,268]. Moreover, PPARγ is able to decrease cyclooxygenase-2 and phospholipase A2-IIA expression and activity, which are responsible for uterine contractions [269].

PPARγ dysregulation by PAEs has been well documented. In silico studies have shown that the PPAR gamma isoform can bind to DIDP, DNDP, MIDP, DINP, MNDP, DNN, DNOP, and MEHP [173,244]. Transactivation studies have indicated that PPARγ is activated by MEHP, MBzP [245], MEHP, MnOP, MINP, and MIDP, and all of these compounds induce adipogenesis [246,270]. Similar results for DINCH and its metabolites were obtained. DINCH does not activate PPARγ, whereas its metabolites induce PPARγ-dependent reporter gene activities in a concentration-dependent manner [126,271]. In the murine trophoblast line HRP-1, DEHP and MEHP exposure induced overexpression of PPARγ transcripts [272]. Similarly, in human placenta, PPARγ expression is increased by DiBP, DBP, and DEHP [234,273,274]. In HTR-8/SVneo cells, PPARγ expression and activity were modulated in a dose-dependent manner. Low MEHP concentrations (0.1 and 1 μM) decreased PPARγ expression and activity, while 10 μM MEHP induced PPARγ expression and activity [85]. Another study conducted on the JEG3 trophoblast cell line showed slightly different results, with an increase in PPARγ activity at 1, 5, and 10 μM MEHP [275], suggesting that phthalate effects vary by compound, cell type and concentration. Regarding cell invasion, MEHP exposure was shown to reduce MMP-9 expression and increase tissue inhibitor of metalloproteinases-1 (TIMP1) via PPARγ activation in HTR-8/SVneo cells [93,219,276]. In primary trophoblast cultures, MEHP induces differentiation with a dose–response effect: a low MEHP concentration promotes differentiation, while a high MEHP concentration inhibits it via PPARγ activation [85]. Despite these approaches, more studies are required to further investigate the effects of DEHP on trophoblastic implantation, invasion, and differentiation. Regarding inflammation, exposure to phthalates, such as DEHP, DBP, DEP, and DnBP, in vitro increases the expression of many pro-inflammatory cytokines, such as TNF-α, IL-6, IL-8, and IL-1, in immune cells [277,278,279], and their expression is inhibited by PPARγ activation [260]. This increase is accompanied by an overexpression of prostaglandins and cyclooxygenase-2 [104]. Finally, an in vitro study on epithelial amniotic cells (Av3) showed that MEHP inhibited PPARγ activation by rosiglitazone, which led to an increase in inflammatory cytokine (IL-6 and IL-8) expression [280]. Few studies have been published on APs and PPARγ. One study showed that exposure to DINCH and MINCH increased PPARγ expression in the human preadipocyte cell lines [281]. Recently, it was established that the anti-inflammatory effect on the amniotic PPARγ is not dysregulated by the alternative plasticizer DINCH and is metabolized to MINCH in human fetal membranes [282]. In conclusion, PAEs decrease trophoblast invasion, proliferation, maturation, and inflammation via the PPARγ pathway. These alterations may lead to placental defects and early weakening of the fetal membranes, which are potential factors for preterm birth. The effects of APs need to be further studied to determine the potential toxicity of these compounds in reproductive tissue and pregnancy outcomes [283]. These investigations could be helped by a new experimental strategy: the four-cell human feto-maternal interface organ-on-chip [284].

### 5.3. Nuclear Receptor Crosstalk at the Feto–Maternal Interface

**Figure 2:**
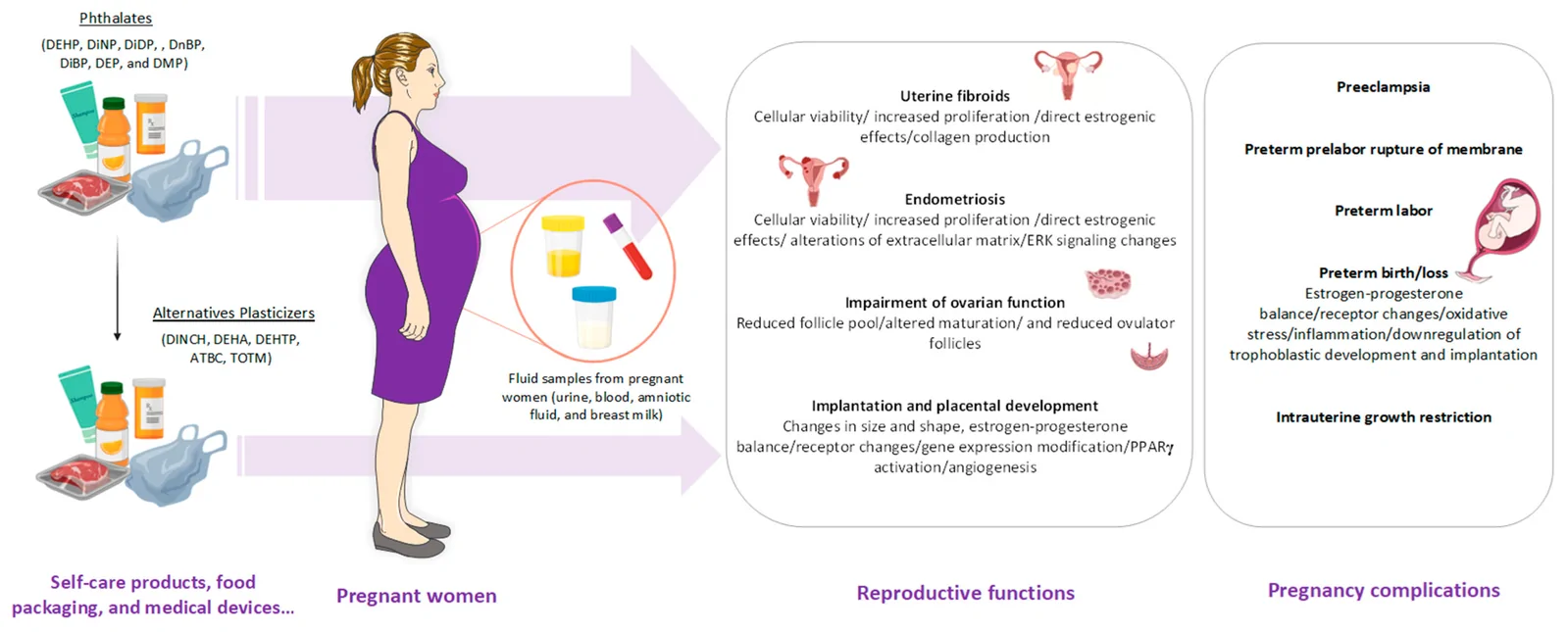
Overview of the effect of phthalates and alternative plasticizers on various reproductive and developmental outcomes.

Although PAEs and APs have been shown to target multiple nuclear receptors, their biological effects may converge through interconnected mechanistic pathways that could collectively contribute to impaired placental function and fetal membranes integrity [126,285,286]. Several hypotheses may be proposed to link nuclear receptor dysregulation with placental and fetal membranes alterations (Figure 2).

Driven by weak agonism or antagonism activity toward steroid receptors (ER, AR, PR, GR and MR) and by modulation of RXR-dependent heterodimers, these plasticizers can dysregulate endocrine signaling essential for placental maturation, trophoblast function and maintenance of uterine quiescence [126,285,286].

Second, inflammatory reprogramming may result from the activation of AhR together with the concomitant inhibition of PPARγ- and GR-mediated anti-inflammatory signaling [260,287,288,289]. In fetal membranes, these alterations could collectively contribute to a shift toward a pro-inflammatory, pro-contractile, and pro-rupture phenotype, potentially mediated by enhanced prostaglandin biosynthesis, cytokine release, and immune cell activation [260,280,287,290,291]. In addition, oxidative stress imbalance may act as an amplifier of receptor dysregulation, which could further enhance inflammatory signaling and contribute to cellular damage in trophoblast and amniotic cells, although these mechanisms remain largely speculative [290,292,293].

Finally, extracellular matrix (ECM) remodeling, driven by altered MMP/TIMP balance, may affect both trophoblast invasion and fetal membrane mechanical stability, two critical determinants of gestational duration [291,294,295].

Alternative plasticizers were introduced as safer replacements for legacy phthalates. However, current evidence suggests that they may still modulate nuclear receptor pathways via nuclear receptor dysregulation [126]. These interactions suggest that structural replacement of phthalates does not fully eliminate endocrine-disrupting potential [288]. In this context, plasticizer exposure could hypothetically contribute to dysregulation of endocrine, metabolic and inflammatory signaling [126,283,289]. However, the functional and biological consequences remain largely unresolved due to the limited number of experimental studies available.

## 6. Perspectives and Conclusions

This review aimed to examine the effects of phthalate and alternative plasticizer exposure on female reproductive health, focusing on ovarian, uterine, and gestational outcomes (Figure 2). It integrates epidemiological evidence of PAE and AP exposure on female fertility and reproductive function. Current knowledge on nuclear receptor–plasticizer interactions is largely derived from in silico studies, which suggest a potential for PAEs and APs to interact with multiple receptor pathways. However, direct experimental evidence from in vivo and in vitro models specifically addressing receptor modulation by plasticizers in the placenta or fetal membranes remains limited.

Some limitations could be attributed to our work. The overarching principle that guides the writing process is to incorporate all relevant papers in order to conduct a comprehensive review of the current state of the art in the bibliography. Unfortunately, it was not possible to incorporate a certain degree of hierarchization across the various papers listed in the manuscript. The subject is extremely novel, and a number of papers dealing with the same subject are currently heterogeneous. This heterogeneity could be explained by a lack of standardization in the experimental process and the heterogeneity of the models used (cellular, animal and human). The studies that are highlighted are those that employ an interesting strategy of using the concentrations found in biological fluids to make the results more relevant to physiological considerations. At present, there is also an imbalance between phthalates and alternative plasticizers in terms of data. This issue could be explained by the fact that alternative plasticizers are a recent development and that the majority of scientific literature focuses on phthalates. Following the prohibition of certain phthalates, the data will be balanced within a few years by the increased use of alternative plasticizers.

Phthalates represent a serious concern for female reproductive health due to their widespread presence and their capacity to interfere with the endocrine system and pregnancy. The listed studies suggest that phthalate exposure may be an important environmental etiological factor contributing to uterine and ovarian disorders, such as polycystic ovary syndrome and endometriosis. Several animal- and cell-based studies support the biological plausibility of associations between PAE and AP exposure and nuclear receptor signaling dysregulation in the placenta and fetal membranes. However, few studies have analyzed the implications of phthalates on nuclear receptors during pregnancy in the context of phthalate exposure.

Phthalates have been reported to decrease steroid hormones, thyroid hormones, glucocorticoids, and retinoic acid expression in the placenta (Figure 1). Nevertheless, the relation between plasticizer exposure and nuclear receptor activity remains insufficiently investigated and requires further investigation to understand mechanisms and their potential effects on the placenta and fetal membranes. Nuclear receptors constitute a large family of transcription factors that regulate developmental and physiological processes by directly controlling gene expression and dysregulation by endocrine disruptors. These receptors are essential for placentation, decidualization, and the maintenance of pregnancy. In this context, this review also discusses potential mechanistic hypotheses arising from the interactions between PAEs and APs and nuclear receptors, particularly hormonal dysregulation, the inflammatory process and oxidative stress, which may collectively contribute to placental and fetal membrane dysfunction.

Additional studies are required to clearly establish the pathways leading to pregnancy disorders caused by phthalates and APs. Recently, new plastic molecules have been developed to replace phthalate compounds in consumer products. However, their effect on female health is not well known, and their potential effects on nuclear receptors in the placenta and fetal membranes remain unclear but seem to be a plausible hypothesis. The study of endocrine-disrupting chemical (EDC) plasticizers has become a growing area in research due to limited knowledge of their action during pregnancy and particularly on fetal membranes. Indeed, fetal membranes are essential tissues for the maintenance of pregnancy and parturition. Initially considered as placental membranes by the scientific and medical community, they are a functionally and regionally distinct tissue, which has been the focus of attention for approximately 30 years. They are clearly associated with serious obstetrical pathologies like premature rupture of membranes, preterm birth and chorioamnionitis. Maintenance of the fetal membranes and potential alteration by EDCs are poorly characterized or understood compared with the placenta, which is a well-studied tissue. The action of phthalates and APs on fetal membranes needs to be determined, with a particular focus on potential alterations in the nuclear receptor pathway. As alternative plasticizers are proposed to replace traditional phthalate ones, understanding the effects and underlying mechanisms of alternative phthalates on reproductive health may improve the development of strategies to reduce the burden of environmental factors on women and their negative consequences on health, fertility, and pregnancy.

## Figures and Tables

**Table 1:** Major phthalates, their corresponding metabolites, and alternative plasticizers studied.

Phthalate					
	Parent Compound	Chemical Formula	Structure	Molecular Weight (g·mol^−1^)	Metabolite
DMP	Dimethyl Phthalate	C_10_H_10_O_4_	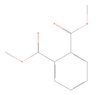	194.2	Monomethyl phthalate MMP
DEP	Diethyl Phthalate	C_12_H_14_O_4_	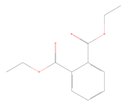	222.2	Monoethyl phthalate MEP
DBP	Di-n-Butyl Phthalate	C_16_H_22_O_4_	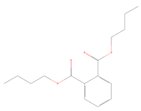	278.4	Mono-n-butyl phthalate MBP
DIBP	Diisobutyl Phthalate	C_16_H_22_O_4_	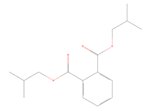	278.4	Mono-iso-butyl phthalate MiBP
BzBP	Butylbenzyl Phthalate	C_19_H_20_O_4_	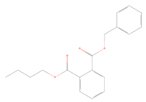	312.4	Monobenzyl phthalate MzBP
DnHP	Di-n-hexyl Phthalate	C_20_H_30_O_4_	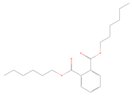	334.4	Mono-n-hexyl phthalate MnHP
DnOP	Di-n-Octyl Phthalate	C_24_H_38_O_4_	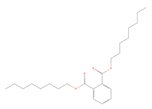	390.6	Mono-3-carboxypropyl phthalate MCPP Mono-n-octyl phthalate MOP
DEHP	Di 2-Ethylhexyl Phthalate	C_24_H_38_O_4_	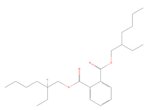	390.6	Mono 2-ethylhexyl phthalate MEHP Mono 2-ethyl-5-hydroxylhexyl phthalate MEHHP Mono 2-ethyl-5-oxohexyl phthalate MEOHP Mono 2-ethyl-5-carboxypentyl phthalate MECPP Mono-2-carboxy-hexyl phthalate MCHP
DnNP	Di-n-nonyl phthalate	C_26_H_42_O_4_	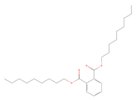	418.6	Mono-n-hexyl phthalate MnHP
DINP	Diisononyl Phthalate	C_26_H_42_O_4_	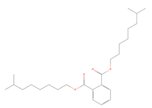	418.6	Mono-iso-nonyl phthtalate MiNP Mono hydroxy-iso-nonyl phthalate MHiNP Mono oxo-iso-nonyl phthalate MIiNP Mono carboxy-iso-ocyl phthalate MCiOP
DIDP	Diisodecyl Phthalate	C_28_H_46_O_4_	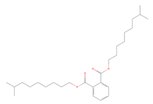	446.7	Mono-isodecyl phthalate MiDP Mono-carboxynonyl phthalate MCNP
DnPeP	Di-n-pentyl phthalate	C_18_H_26_O_4_	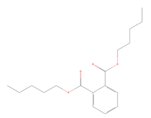	306.4	Mono-n-pentyl phthalate mnPeP
**Alternative plasticizers**					
ATBC	Acetyl tributyl citrate	C_20_H_34_O_8_	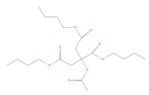	402.5	Tri-butyl citrate TBC Acetyl di-butyl citrate ADBC
DINCH	Diisononyl 1,2-cyclohexanedicarboxylic acid	C_26_H_48_O_4_	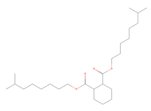	424.7	Cyclohexane-1,2-dicarboxylic acid-mono-4-methyl-octyl ester MINCH cyclohexane-1,2-dicarboxylic acid-mono-(7-hydroxy-4-methyl)-octyl ester OH-MINCH cyclohexane-1,2-dicarboxylic acid-mono-(7-oxo-4-methyl)-octyl ester oxo-MINCH cyclohexane-1,2-dicarboxylic acid-mono-(7-carboxylate-4-methyl)-heptyl ester cx-MINCH cyclohexane-1,2-dicarboxylic acid-mono-2-nonyl ester (M2NCH)
DEHA	Bis(2-ethylhexyl) adipate	C_22_H_42_O_4_	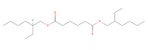	370.6	mono-2-ethyl-5-hydroxyhexyl adipate 5OH-MEHA mono-2-ethyl-5-oxohexyl adipate 5oxo-MEHA mono-5-carboxy-2-ethylpentyl adipate (5cx-MEPA)
DOTP/DEHTP	Dioctyl Terephthalate	C_24_H_38_O_4_	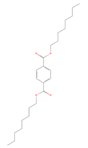	390.6	mono-2-ethyl-5-carboxypentyl terephthalate MECPTP mono-2-ethyl-5-hydroxyhexyl terephthalate MEHHTP
TOTM	Tris(2-ethylhexyl) trimellitate	C_33_H_54_O_6_	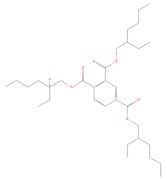	546.8	1,2-di-(2-ethylhexyl) trimellitate (1,2-DEHTM) 1,4-di-(2-ethylhexyl) trimellitate (1,4-DEHTM) 2,4-di-(2-ethylhexyl) trimellitate (2,4-DEHTM) 1-mono-(2-ethylhexyl) trimellitate (1-MEHTM) 2-mono-(2-ethylhexyl) trimellitate (2-MEHTM) 4-mono-(2-ethylhexyl) trimellitate (4-MEHTM)

**Table 2:** Comparative interactions of phthalates (PAEs) and alternative plasticizers (APs) with nuclear receptors.

Receptor	Interaction with PAEs	Interaction with APs
Estrogen receptors (ER)	++	+
Androgen receptor (AR)	++	+
Progesterone receptor (PR)	+	NA
Glucocorticoid receptor (GR)	++	+
Mineralocorticoid receptor (MR)	+	NA
Retinoic acid receptor (RAR) Retinoid X receptor (/RXR)	++	+
Liver X receptor (LXR)	+	NA
Thyroid hormone receptor (TR)	++	+
Constitutive androstane receptor (CAR)	++	NA
Pregnane X receptor (PXR)	++	NA
Aryl hydrocarbon receptor (AhR)	++	+
Peroxisome proliferator-activated receptor (PPAR)	++	+

This table summarizes the available evidence on interactions between PAEs or APs and nuclear receptors involved in reproductive regulation. Notes: “+” limited or emerging evidence, based on a smaller number of compounds and in silico predictions; “++”well-established evidence supported by multiple experimental studies and consistent in silico and in vitro findings demonstrating receptor interactions or modulation by several compounds; NA indicates no available data reported in the reviewed literature regarding interactions between the receptor and the plasticizers.

## Data Availability

No new data were created or analyzed in this study. Data sharing is not applicable to this article.

## Abbreviations

The following abbreviations are used in this manuscript:

AF	amniotic fluid
AhR	aryl hydrocarbon receptor
AP	alternative plasticizers
AR	androgen receptor
BBzP	butyl benzyl phthalate
CAR	constitutive androstane receptor
DBP:	di-butyl phthalate
DEP:	di-ethyl phthalate
DEHA	di-2-ethylhexyl adipate
DEHP	di-hexyl phthalate
DiDP	di-isodecyl phthalate
DINCH	diisononyl cyclohexane-1,2-dicarboxylate
DiNP	di-isononyl phthalate
EDC	endocrine disruptor chemicals
ER	estrogen receptor
GR	glucocorticoid receptor
HMW	high molecular weight
LMW	low molecular weight
LXR	liver X receptor
MBzP	mono-benzyl phthalate
mCMHP	mono-(2-carboxymethyl-hexyl) phthalate
MECPP	mono-(2-ethyl-5-carboxy-pentyl) phthalate
MEHHP	mono-(2-ethyl-5-hydroxy-hexyl) phthalate
MEHP	mono(2-ethylhexyl) phthalate
MEOHP	mono-(2-ethyl-5-oxo-hexyl) phthalate
MEP	mono-ethyl phthalate
MIBP	mono-isobutyl phthalate
MiDP	mono-isodecyl phthalate
MMP	mono-methyl phthalates
MR	mineraloid receptor
PAE	phthalates esters
PPAR	peroxisome proliferator-activated receptor
PR	progesterone receptor
PXR	pregnane X receptor
RAR	retinoic acid receptor
RXR	retinoid X receptor
TR	thyroid receptor
